# Kudiezi injection mitigates myocardial injury induced by acute cerebral ischemia in rats

**DOI:** 10.1186/s12906-016-1514-1

**Published:** 2017-01-05

**Authors:** Xuemei Liu, Ye Tao, Fengli Wang, Ting Yao, Chen Fu, Hong Zheng, Yan Yan, Xiao Liang, Xiangning Jiang, Yunling Zhang

**Affiliations:** 1 Central Laboratory, Dongfang Hospital, Beijing University of Chinese Medicine, 6 Zone One of Fangxingyuan, Fang Zhuang, Fengtai District, Beijing, 100078 China; 2 Patent Examination Coperation Center of the Patent Office, SIPO, Beijing, 100190 China; 3 China-Japan Friendship Hospital, 2 YinghuaDongjie, Hepingli, Beijing, 100029 China; 4 Department of Pediatrics, University of California, San Francisco, 675 Nelson Rising Lane Room 494, San Francisco, CA 94158 USA

**Keywords:** Cerebral ischemia, Myocardial injury, Oxidative stress, Mitochondria, Kudiezi injection

## Abstract

**Background:**

Kudiezi (KDZ) injection is commonly used in traditional Chinese medicine as treatment for cerebral infarction and angina pectoris. The present study investigated the therapeutic effects of KDZ injection on myocardial injury induced by acute cerebral ischemia and the possibly protective mechanisms.

**Methods:**

Rats were divided into three groups: sham, 6h-ischemia, and KDZ treatment (KDZ). The neurological deficits were determined by the Garcia score. The cerebral infarct volume was measured by 2,3,5-triphenyltetrazolium chloride (TTC) staining, and brain water content was also evaluated. Serum creatinine kinase (CK), lactate dehydrogenase (LDH), and creatine kinase-myocardial band (CK-MB) activity, myocardial tissue malondialdehyde (MDA) levels, L-Glutathione (GSH) levels, and superoxide dismutase (SOD) activity as well as mitochondrial cytochrome c oxidase (COX) activity were determined. Mitochondrial COX I and COX III mRNA expressions of myocardial tissues were measured by RT-PCR.

**Results:**

Impaired neurological function and brain edema were observed in the 6h-ischemia group. TTC staining showed that the 6h-ischemia group had larger infarct zones than the sham group. Myocardial ischemic changes (widened myocardial cell gap, cracks, and obvious edema) were detected in the 6h-ischemia group compared with the sham group, with elevated serum CK-MB activity and CK and LDH levels. Electrocardiography showed lower medium frequency (MF) and high frequency (HF) in the 6h-ischemia group compared with the sham group. In myocardial tissue, COX activity was elevated in the 6h-ischemia compared with the sham group, while SOD, GSH, and MDA levels, and COX I and COX III mRNA expressions remained unchanged. KDZ injection decreased neurological impairment, brain edema, gaps between cells, and infarct size. Compared with the 6h-ischemia group, it reduced serum CK-MB activity and CK and LDH levels, and MDA levels in myocardial tissue. KDZ significantly increased GSH levels, SOD activity, and mitochondria COX activity and the expression of COX I and COX III mRNA in myocardial tissue compared with the sham group.

**Conclusion:**

KDZ injection had a protective effect against cerebral ischemia in rats. KDZ injection could also alleviate myocardial injury after acute cerebral ischemia in rats. The possible mechanisms involve the regulation of the oxidative stress/antioxidant capacity after cerebral ischemia.

## Background

The most common cause of brain damage is hypoxia-ischemia [1]. Focal brain ischemia or stroke is a leading cause of death and adult disability. Although improving the neurological symptoms is the primary goal of stroke intervention, myocardial injury can occur during the acute phase and the recovery period after a stroke. Stroke-associated cardiac complications such as heart attack, congestive heart failure, abnormal heart rhythms, and cardiac arrest, may also lead to death [2, 3]. Of note, 2–6% of deaths are of cardiac origins in the first 3 months following ischemic stroke [3, 4]. It is also an indication of an unfavorable prognosis in future stages [5, 6]. Current clinical trials and animal experiments studying myocardial injury induced by cerebrovascular diseases mainly focused on cerebral hemorrhage and subarachnoid hemorrhage and rarely on cerebral ischemia. A rodent model is needed to investigate the myocardial injuries secondary to cerebrovascular diseases and underlying mechanisms.

Oxidative stress plays an important role in the pathogenesis of ischemic stroke. The formation of reactive oxygen species (ROS) and free radicals induce damage [7]. Oxidative stress is an imbalance between the production of ROS and antioxidant factors of cells [8]. Oxidative stress has been shown to increase the levels of lipid peroxidation, which may have a role in myocardial tissue damage associated with acute cerebral ischemia [9]. Cytochrome c oxidase (COX) is the key enzyme in mitochondrial oxidative metabolism and is the terminal electron transport oxidase in the mitochondrial respiratory chain. The association of oxidative stress and mitochondria was demonstrated by the up-regulation of several COX genes involved in cellular oxidative stress [10]. COX is a crucial component of the mitochondrial respiratory chain and is of outmost importance for providing cellular energy [11]. Increased COX activity has been associated with increased oxidative stress [10, 12], but COX inhibition was also found to increase the susceptibility to oxidative stress and to accelerate mitochondrial apoptosis in response to oxidative stress [13].

Kudiezi (KDZ) injection extracted from the Chinese herb *Ixeris denticulate*is one of many agents used in traditional Chinese medicine for the treatment of ischemic stroke and myocardial infarction. It is approved by the Chinese National Drug Administration. A study showed that KDZ injection significantly reduced the degree of neurological impairment (National Institutes of Health Stroke Score (NIHSS)) and improved activities of daily living (ADL) score in 166 patients with ischemic stroke [14]. Zhou et al. [15] showed in 180 patients with acute cerebral infarction that KDZ injection can reduce serum highly-sensitive C-reactive protein (hs-CRP) and interleukin (IL)-18 of patients with acute cerebral infarction. Liu et al. [16] showed that KDZ injection can reduce serum IL-6 and tumor necrosis factor (TNF)-α of patients with acute cerebral infarction. These previous studies also suggest that KDZ injection can improve patient quality of life. Pharmacological studies have shown that KDZ injection can reduce IL-1β, TNF-α, and other inflammatory cytokine expression, down-regulate the protein expression of TLR4 and NOX4 (inflammatory signaling pathways), improve the status of the middle cerebral artery occlusion (MCAO) rat model of pathological infarction, reduce cerebral infarct size, can regulate inflammatory responses, and has neuroprotective effects in cerebral ischemia in rats [17, 18]. In vitro experiments using human brain microvascular endothelial cells showed that KDZ injection can downregulate the expression of intercellular adhesion molecule (ICAM)-1 and vascular cell adhesion molecule (VCAM)-1 induced by high glucose injury [19].

Nevertheless, the effects and mechanisms of KDZ injection on myocardial injury after cerebral ischemia are still unclear. In the present study, a rat model of acute cerebral ischemia was established by MCAO. The therapeutic effect of KDZ injection on both brain and heart were investigated, as well as the protective mechanisms.

## Methods

### KDZ injection

**Fig. 1:**
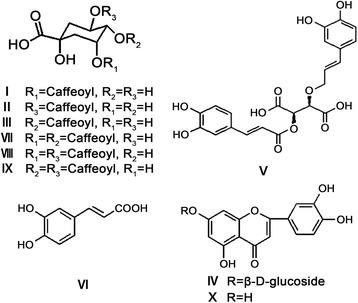
The structures of ten active components of Kudiezi (KDZ) injection

KDZ injection was obtained from TonghuaHuaxia Pharmaceutical Co. Ltd. (Jilin, China; certification number: Z20025450). The ten active components of the KDZ injection are shown in Fig. 1.

### Animals and grouping

Male Sprague Dawley rats (8-week old, 220–240 g) were obtained from Vital River Co. (Beijing, China; certification number: SCXK (Jing): 2012–0001). All animals received humane care in compliance with the Dongfang Hospital, Beijing University of Chinese Medicine guidelines and the research council’s criteria. Animal procedures were approved by the institutional animal care and use committee of Dongfang Hospital, Beijing University of Chinese Medicine. The animals were allowed to acclimatize for 5 days before experiments.

They were randomly divided into three groups: sham, 6h-ischemia, and KDZ treatment (KDZ). The rats in the sham groups were subjected to surgical operation without MCAO. In the 6h-ischemic and KDZ groups, rats were subjected to MCAO on the left hemisphere. The animals in the KDZ group were treated with KDZ injection (40 ml of KDZ injection mixed with 250 ml of 0.9% sodium chloride) intraperitoneally injected at 22.65 ml/kg within 10 min after MCAO. The sham and 6h-ischemia groups received 0.9% sodium chloride at 1 ml/100 g by intraperitonealinjection.

### Middle cerebral artery occlusion

Rats were anesthetized with 10% chloral hydrate (3.5 ml/kg body weight) intraperitoneally. Cerebral ischemia was conducted according to the method by Longa et al. [20, 21] with some modifications. A 24-mm nylon monofilament (Cinontech Limited Company, Beijing, China) was inserted into the internal carotid artery through a small incision on the left common carotid artery and advanced 18 ± 2 mm to occlude the middle cerebral artery. The filament was left in place until sacrifice at 6 h after occlusion (without reperfusion). Rats in the sham group underwent the same procedure without monofilament insertion.

### Assessment of neurological function

Neurological deficit scoring was performed by two investigators blind to grouping at 6 h after MCAO. The neurobehavioral scoring was performed according to the methods of Garcia [22]: spontaneous activity (in cage for 5 min); symmetry of movements (four limbs); symmetry of forelimbs (outstretching while held by tail); climbing wall of wire cage; reaction to touch on either side of trunk; and response to vibrissae touch. Each test is scored 0–3 points. The total scores range 3–18. The lower the score, the more severe is the neurological impairment.

### Heart rate variability (HRV)

After assessment of the neurological function, rats were anesthetized with 10% chloral hydrate (3.5 ml/kg body weight) intraperitoneally. The rats were implanted with needle electrode connected with a MP100 physiography device (Biopac Systems Inc., Goleta, CA, USA) and the device recorded the electrocardiographic signals for 120 s, which were subsequently used to derive R-R intervals. Spectral power of HRV was obtained by applying the fast Fourier transformation (FFT) and ranged from 0 to 3 Hz. The low frequency band (LF) was integrated over 0.04–0.20 Hz, the medium frequency band (MF) was integrated over 0.21–0.60 Hz, and the high frequency band (HF) was integrated over 0.61–3.0 Hz. The MF/LF ratio was also calculated.

### Blood sample and tissue collection

After the 6-h KDZ treatment, the rats were anesthetized with 10% chloral hydrate (3.5 ml/kg body weight). Blood was collected from the abdominal aorta. The serum was rapidly separated from blood and processed for determination of serum creatinine kinase (CK), lactate dehydrogenase (LDH), and creatine kinase-myocardial band (CK-MB) activity. The brain cortex and myocardial tissues were dissected. Changes in cortex weight were recorded to determine brain edema.

### Measurement of ischemic infarction volume and brain water content

Coronal sections (2 mm) were immersed in 1% 2,3,5-triphenyltetrazolium chloride (TTC) in phosphate-buffer saline (pH 7.4) at 37 °C for 15 min and then fixed with 10% paraformaldehyde for 10 min [5]. The white color represented infarct tissue and the red color represented normal tissue. TTC-stained sections were photographed and the images were analyzed using Image-Pro Plus 6.0 (Olympus, Tokyo, Japan) to calculate the infarct volume. The lesion volumes were calculated as a percentage of the contralateral hemisphere volume to compensate for the effect of brain edema using the following formula: [total infarct volume-(volume of intact ipsilateral hemisphere-volume of intact contralateral hemisphere)]/contralateral hemisphere volume × 100% [6].

The brain water content was measured using the standard wet dry method. A brain slice was cut and the slice was divided into the ipsilateral and contralateral hemispheres. The two hemisphere slices were immediately weighed to obtain the wet weight, dried for 24 h in an oven at 100 °C, and then reweighed to obtain the dry weight. Brain water content was calculated as a percentage using the following formula: [(wet weight-dry weight)/wet weight] × 100%.

### Serum CK, LDH, and CK-MB activity

Blood taken from the abdominal aorta was centrifuged at 3500 rpm at 4 °C in a refrigerated centrifuge for 10 min. Supernatant was used for ELISA assays [Rat LDH ELISA Kit, Enzyme-linked Biotechnology Co., Ltd., Shanghai, China; Rat CK ELISA Kit, Lianshuo Biological Technology Co., Ltd., Shanghai, China; CK-MB activity Kit, RB, USA] and measured at 450 nm with a microplate reader (RB, USA) according to the manufacture’s protocol. The concentrations of CK-MB, CK, and LDH was calculated and expressed as U/L.

### Histological changes

Paraffin serial sections (5 μm) were prepared from cortex and myocardial tissues. Sections were de-waxed and rehydrated. Endogenous peroxidase was quenched for 20 min using 3% (v/v) hydrogen peroxide in phosphate-buffered saline (PBS). Staining was performed using H&E and hematoxylin basic fuchsin picric (HBFP). Histological images were captured with a BX71 microscope (Olympus, Tokyo, Japan).

### Assessment of SOD activity, MDA content, and GSH content in myocardial tissues

Myocardial tissues were rinsed, weighed, and homogenized with nine volumes of 0.9% normal saline for 10 min. After centrifugation at 3000 rpm for ten minutes at 4 °C, the supernatants were collected and used immediately. The levels of MDA were determined by the TBA method, the levels of GSH were determined by a colorimetric method, and the activity of SOD was measured by the hydroxylamine method, all using commercial kits (Jiancheng Limited Company, Jiangsu, China).

### Mitochondria isolation of myocardial tissue

Minced heart tissues were homogenized in MESH-BSA (15 mL/g tissue). The homogenates were centrifuged at 1500 *g* for 5 min to remove cell debris and the nuclear fraction. The supernatant was further centrifuged at 13,000 × *g* for 10 min. The pellet was resuspended in 20 mL of MESH buffer and centrifuged at 13,000 × *g* for 10 min. The mitochondria pellet was suspended in 0.5 mL of MESH per g of tissue to yield a protein concentration of 25 mg/mL, according to the manufacturer’s instructions (Tissue mitochondria isolation kit, Biosea Biotechnology Limited Company, Beijing, China).

### Measurement of COX activity in myocardial mitochondria

COX activity in mitochondria of myocardial tissues was measured at 550 nm using the reduced cytochrome c method, according to the manufacturer’s instructions (COX activity kit, Jiemei Gene Limited Company, Shanghai, China).

### Semi-quantitative RT-PCR analysis

Total mitochondrial RNA was isolated from myocardial tissues using Trizol (Invitrogen Inc., Carlsbad, CA, USA), and then reverse-transcribed using the RT-PCR ReverTra-Plus^TM^ kit (TOYOBO Co., Ltd, Tokyo, Japan) according to the manufacturer’s instructions. Semi-quantitative RT-PCR analysis of COX I and COX III mRNA expressions was performed using the Prime Script RT-PCR kit (Clontech Laboratories Inc., Mountain View, CA, USA) according to the manufacturer’s instructions and a PCR system (AG-22331; Eppendorf, Hamburg, Germany). Glyceraldehyde-3-phosphatedehydrogenase (GAPDH) was used as control. The primers were: COX I: forward: 5′-GGC TTC GGG AAC TGA CTT GT-3′, reverse: 5′-AAG GAT TGG GTC TCC ACC TC-3′ (annealing temperature: 60 °C; 30 cycles; PCR product: 462 bp); COX III: forward: 5′-GCC ACC ACA CCC CTA TTG TA-3′, reverse: 5′-TCC CGT TGC TAT GAA GAA TG-3′ (annealing temperature: 60 °C; 30 cycles; PCR product: 401 bp), and GAPDH: forward: 5′-TGT TCC TAC CCC CAA TGT GT-3′, reverse: 5′-CCC TGT TGC TGT AGC CGT AT-3′ (annealing temperature: 60 °C; 30 cycles; PCR product: 401 bp). The relative expression of mRNA of a specific gene to the internal control was calculated as the ratio of relative optical density using a gel imaging analyzer (EPS301; GE Healthcare, Waukesha, WI, USA) with gel analysis software (GE Healthcare, Waukesha, WI, USA).

### Statistical analysis

All data were expressed as mean ± standard error of the mean (SEM) and analyzed using the independent sample *t* test (for infarct volume and brain edema) or one-way analysis of variance (ANOVA) with the least significant difference (LSD) as post hoc test (for all other analyses), as appropriate. All analyses were performed with SPSS 17.0 (IBM, Armonk, NY, USA). Two-sided *P*-values < 0.05 were considered statistically significant.

## Results

### KDZ injection improved neurological function in a focal cerebral ischemia rat model

**Fig. 2:**
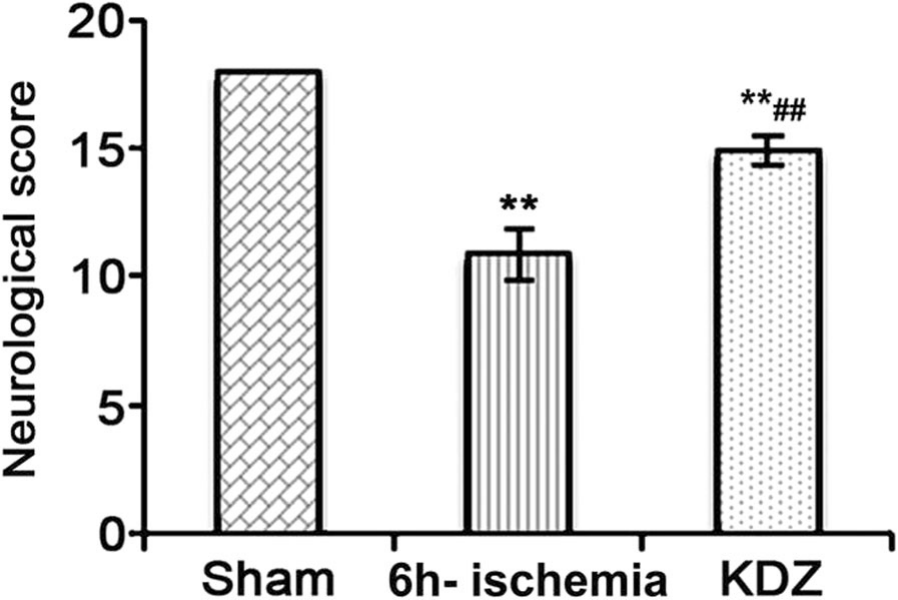
Effects of KDZ injection on the neurological function of rat models of focal cerebral ischemia Sham: rats were subjected to surgical operation without occlusion of the right middle cerebral artery (MCAO) and without KDZ treatment; 6h-ischemia: rats were subjected to MCAO without KDZ treatment; and KDZ: rats were subjected to MCAO and KDZ treatment. Neurological impairment was evaluated using the Garcia score. Data are shown as mean ± standard error of the mean (SEM) (*n* = 8/group). **P < 0.01 *vs.* sham; ##*P* < 0.01 *vs.* 6h-ischemia

The rats in sham group could walk in a straight line and had a steady gait, both forelimbs could unbend, and there was no neurological damage. Garcia scores were about 18. The rats in the 6h-ischemia group walked slowly and failed to flex their left forelimb fully. The rats could not walk straightly and leaned to the left. The Garcia scores in the 6h-ischemia group were lower than sham group (7.50 ± 1.41, *P* < 0.01). Moreover, the Garcia cores in the KDZ group were significantly higher than those in the 6h-ischemia group despite that it was still lower than in the sham group (10.25 + 1.28, both *P* < 0.01) (Fig. 2).

### KDZ injection reduced cerebral infarct volume and brain water content in the focal cerebral ischemia rat model

**Fig. 3:**
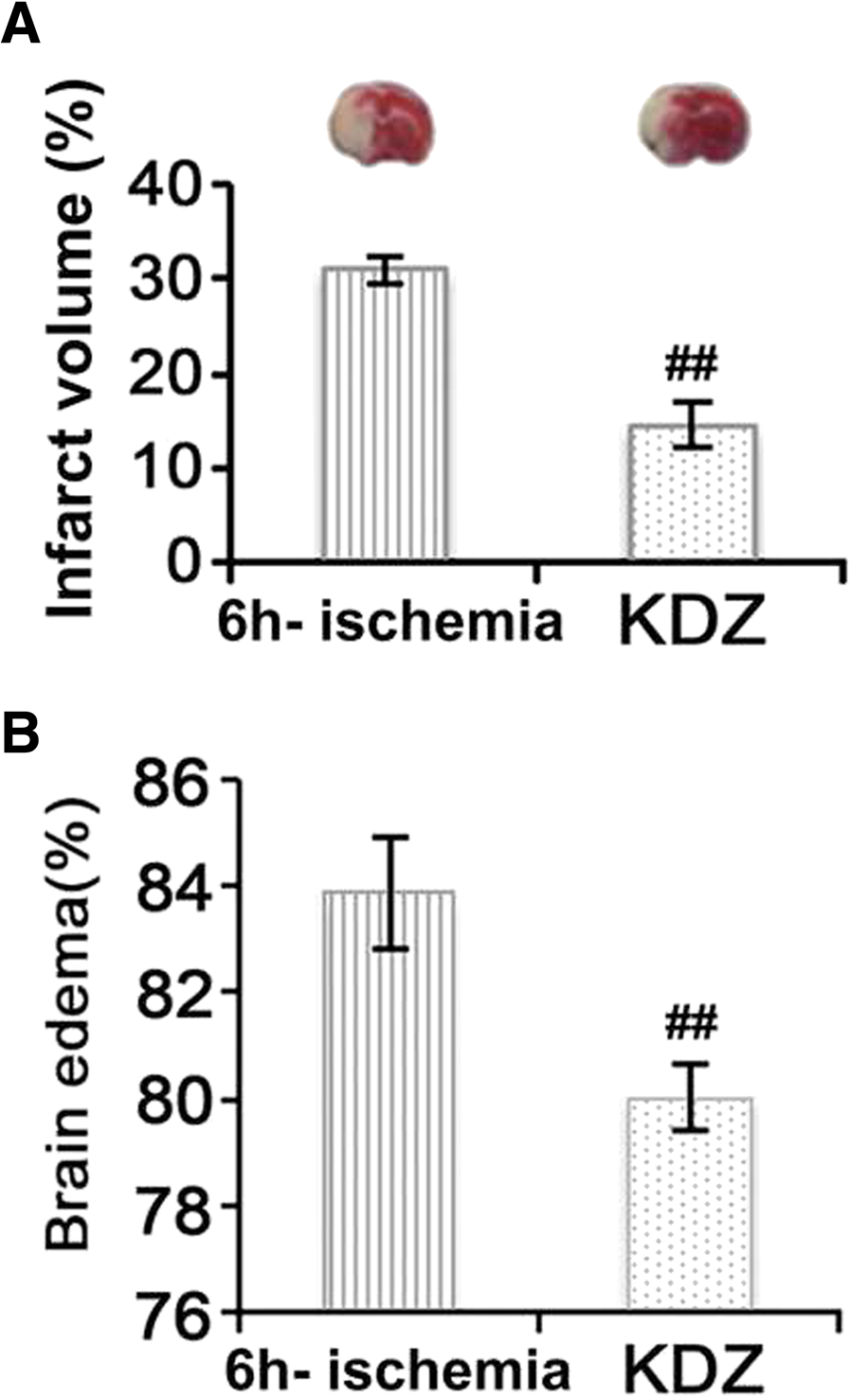
Effects of KDZ injection on the infarct volume and brain edema of rat models of focal cerebral ischemia. **a** Infarct volume was determined by 2,3,5-triphenyltetrazolium chloride staining shown as white color. Infarct volume (%) in the 6h-ischemia and KDZ groups was quantitatively evaluated. **b** Brain edema (%) in the 6h-ischemia and KDZ groups. Data are shown as mean ± SEM (*n* = 8/group). ##*P* < 0.01 *vs.* 6h-ischemia

Brain infarct volume was measured with TTC staining. Rats in the sham group had no ischemic damage. Rats in the 6h-ischemia group had an infarct volume of 31.01 ± 1.43%, whereas KDZ treatment decreased the volume to 14.57 ± 2.41% (*P* < 0.01; Fig. 3a). In the 6h-ischemia group, cerebral brain water content was 83.83 ± 1.06%, whereas in the KDZ group, it was lowered to 80.06 ± 0.38% (*P* < 0.01; Fig. 3b).

### KDZ injection decreased CK-MB activity, and CK and LDH levels in serumin the focal cerebral ischemia rat model

**Fig. 4:**
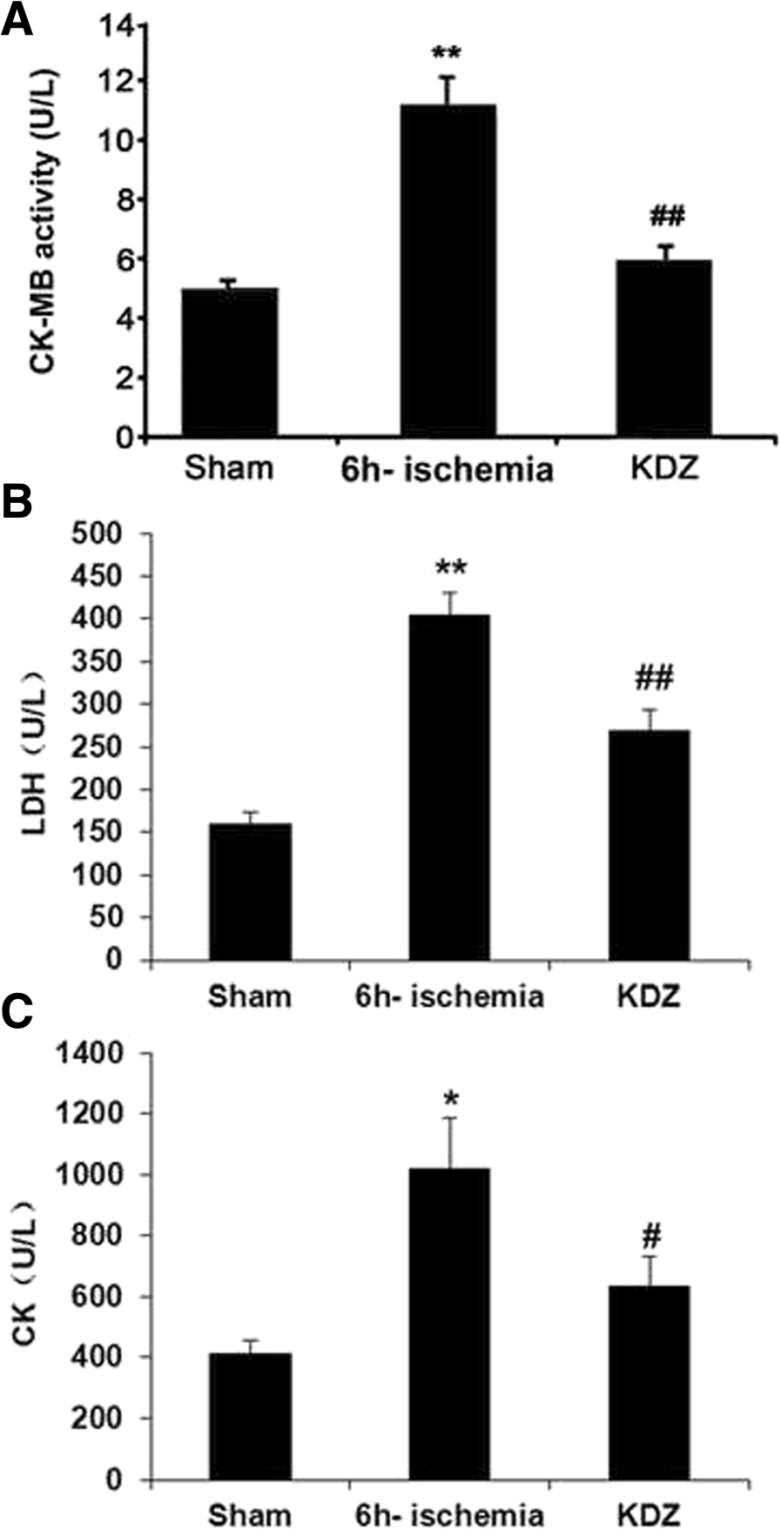
Effects of KDZ injection on serum creatinine kinase (CK), lactate dehydrogenase (LDH), and creatine kinase-myocardial band (CK-MB) activity of rat models of focal cerebral ischemia. **a** CK-MB activity, **b** CK, and **c** LDH were determined by ELISA. Data are shown as mean ± SEM (*n* = 10/group for CK-MB activity; *n* = 12/group for CK and LDH). *P < 0.05, **P < 0.01 *vs.* sham; #*P* < 0.05, ##*P* < 0.01 *vs.* 6h-ischemia

We measured the CK, LDH, and CK-MB activity in the serum of the rats as indications of myocardial damage or infarction. CK-MB activity was 5.01 ± 0.29 U/L in the sham group, and was increased to 11.01 ± 1.14 U/L at 6 h post-ischemia (*P* < 0.01 *vs.* the sham group), whereas it was declined to 5.87 ± 0.54 U/L in the KDZ group (*P* < 0.01 *vs.* the 6h-ischemia group) (Fig. 4a). LDH levels were 160.0 ± 13.7 U/L in the sham group, and was increased to 404.8 ± 26.1 U/L at 6 h post-ischemia (*P* < 0.01 *vs.* the sham group), whereas it was declined to 269.4 ± 23.4 U/L in the KDZ group (*P* < 0.01 *vs.* the 6h-ischemia group) (Fig. 4b). CK levels were 409.8 ± 46.0 U/L in the sham group, and was increased to 1019.5 ± 165.8 U/L at 6 h post-ischemia (*P* < 0.01 *vs.* the sham group), whereas it was declined to 633.0 ± 101.7 U/L in the KDZ group (*P* < 0.05 *vs.* the 6h-ischemia group) (Fig. 4c).

### KDZ injection alleviated the myocardial ischemic changes in the focal cerebral ischemia rat model

**Fig. 5:**
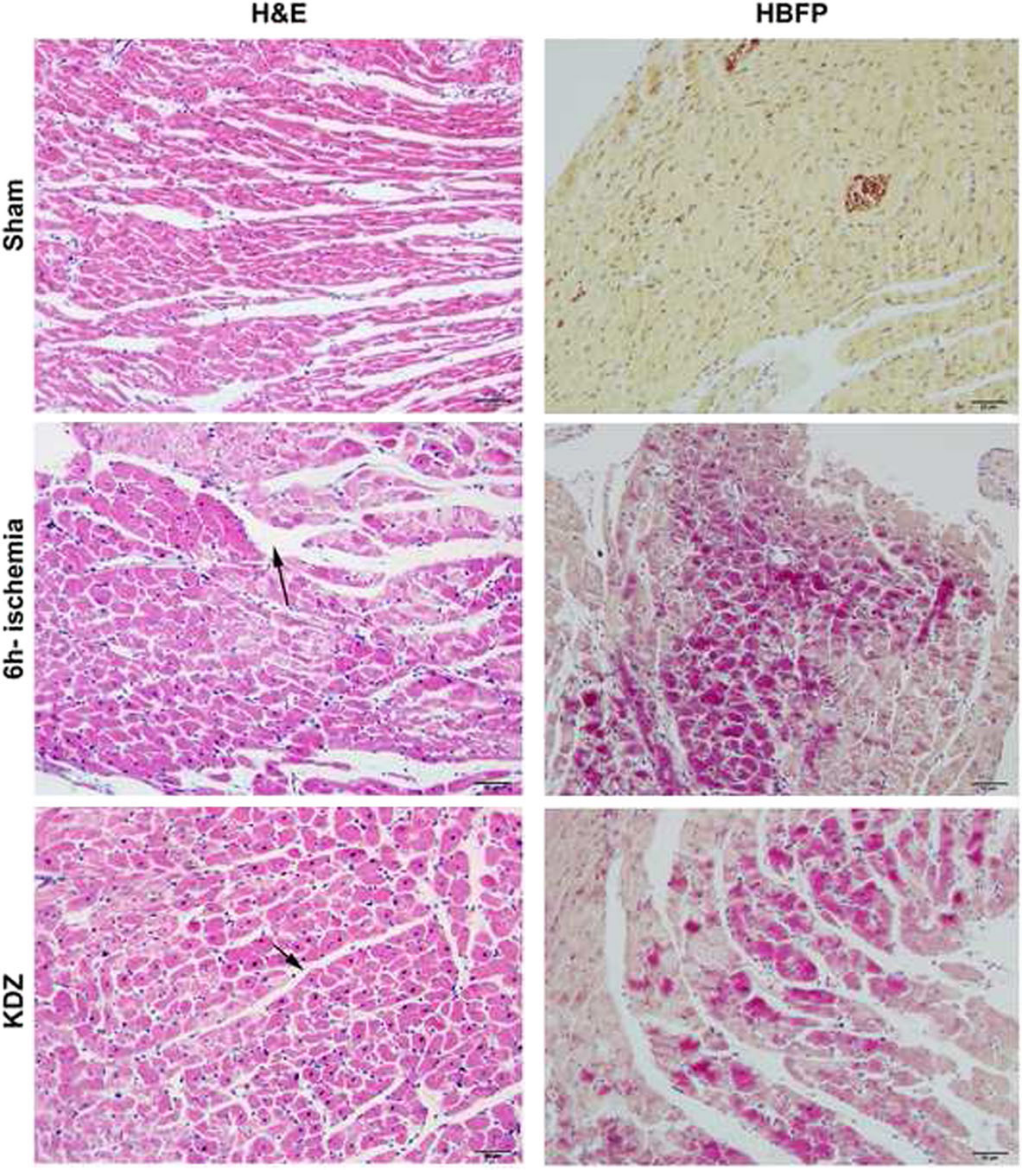
Effects of KDZ injection on changes of myocardial histomorphology of rats models of acute cerebral ischemia by H&E staining and hematoxylin-basic fuchsin picric (HBFP) staining (magnification: ×200). In the sham group, H&E staining showed that cardiomyocyte morphology was normal, myofibrils were orderly arranged, and myocardial cell gaps were uniform. In the 6h-ischemia group, myofibril arrangement was disorderly, myocardial cell gaps were widened, cracks had appeared, and edema was obvious. In the KDZ group, myocardial cell edema was decreased and gaps were smaller. Arrows: gaps. In the sham group, HBFP staining showed that myocardial fibers were golden and the nuclei were blue. In the 6h-ischemia group, myocardial fibers were red and arranged in large sheets with diffuse distribution and dark red staining. In the KDZ group, compared with the model group, red staining of myocardium fiberswas weaker, with a smaller number, and more dispersed

Myocardial ischemic changes (widened myocardial cell gap, cracks, and obvious edema) were detected by H&E staining 6h after MCAO (Fig. 5). These lesions were alleviated in the KDZ group, as shown by lower edema of myocardial cells and smaller gaps (Fig. 5). HBFP staining is a non-enzymatic histochemical technique to detect early myocardial ischemia [23]. Myocardial ischemia was observed in the endocardial myocardium and musculi papillary, and the middle of the ventricular wall at 6h after cerebral ischemia. Myocardial ischemia was alleviated in the KDZ group compared with the 6h-ischemia group (Fig. 5).

### KDZ injection increased MF and HF in the focal cerebral ischemia rat model

HRV is of substantial importance to evaluate autonomic control mechanisms and to identify patients with an increased risk of myocardial mortality [24]. HRV is mainly composed of LF, MF, and HF. MF is affected by both sympathetic and parasympathetic myocardial activities. HF is a quantitative indicator for the detection of myocardial parasympathetic activity. The MF/HF ratio reflects the balance of sympathetic and parasympathetic myocardial activity. In the 6h-ischemia group, MF and HF were lower compared with the sham group (both *P* < 0.05). After KDZ treatment, MF and HF were higher than in the 6h-ischemia group (both *P* < 0.05) (Table 1).

**Table 1:** Effect of Kudiezi injection on heart rate variability in rat models of focal cerebral ischemia

Group	LF (S^2^/Hz)	MF (S^2^/Hz)	HF (S^2^/Hz)	MF/HF
Sham	0.0183 ± 0.0032	0.070 ± 0.0124	0.0230 ± 0.0041	3.017 ± 0.004
6h-ischemia	0.0166 ± 0.0035	0.059 ± 0.0125^*^	0.0194 ± 0.0036^*^	3.023 ± 0.006
KDZ	0.0162 ± 0.040	0.064 ± 0.0128^#^	0.0212 ± 0.0040^#^	3.018 ± 0.007

Data are shown as mean ± standard error of the mean (SEM) (*n* = 12/group)

*LF* low frequency band, *MF* middle frequency band, *HF* high frequency band, *KDZ* Kudiezi injection

*P < 0.05 *vs.* sham group; ^#^ *P* < 0.05 *vs.* the 6h-ischemic group

### KDZ injection increased SOD activity and GSH content and decreased MDA content in myocardial tissue after brain ischemia

**Fig. 6:**
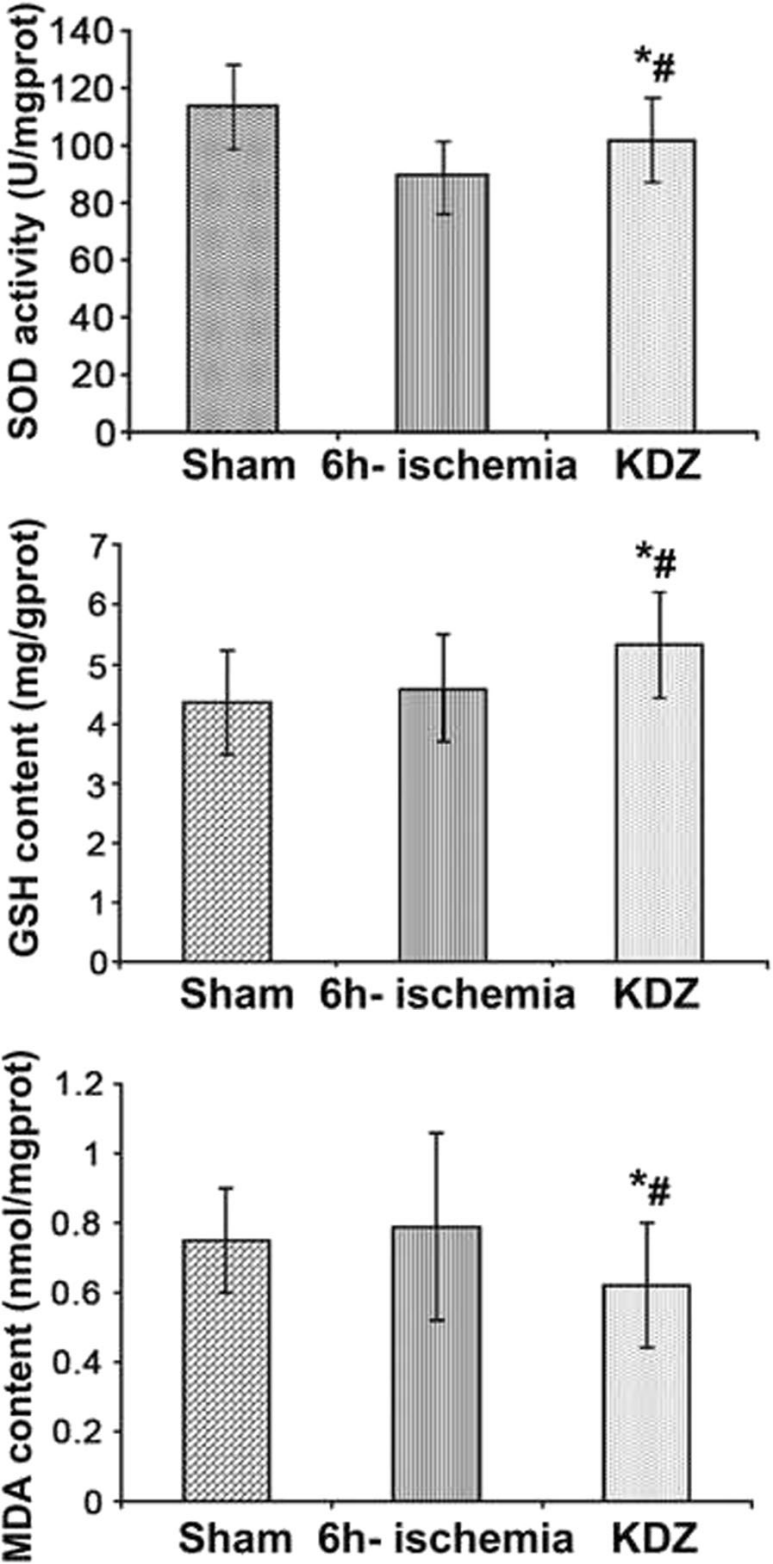
Effects of KDZ injection on SOD activity and MDA and GSH content in myocardial tissues of rat models of focal cerebral ischemia. Data are shown as mean ± SEM (n = 12/group). *P < 0.05 *vs.* sham; #*P* < 0.05 *vs.* 6h-ischemia

There was no difference in the levels of SOD activity, GSH, and MDA contents in myocardial tissues in the 6h-ischemia group compared with the sham group (all *P* > 0.05). Compared with the 6h-ischemia group, SOD activity and GSH content were significantly increased (both *P* < 0.05) while the MDA levels were reduced in the KDZ group (*P* < 0.05) (Fig. 6).

### KDZ injection has no effect on COX activity in the mitochondria of myocardial tissues after brain ischemia

**Fig. 7:**
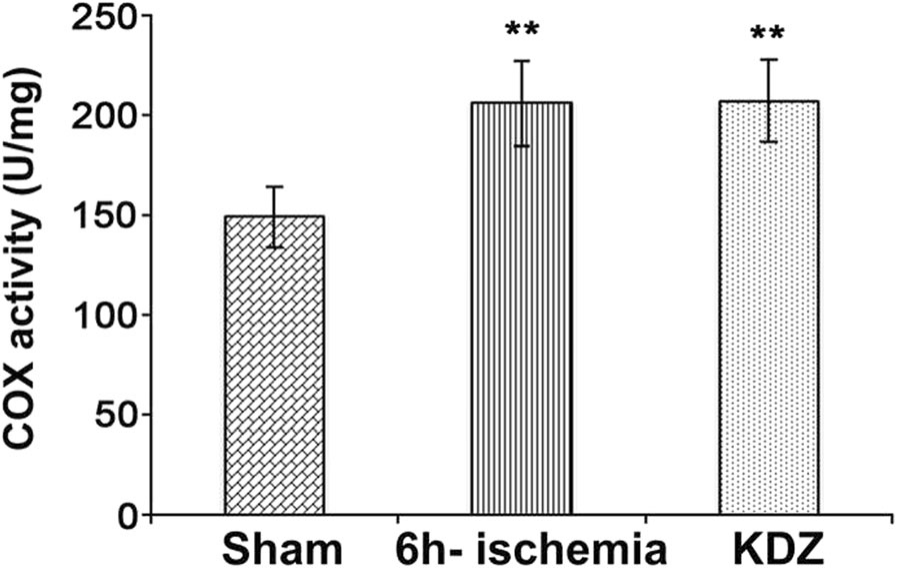
Effect of KDZ injection on mitochondrial COX activity in myocardial tissues of rat models of focal cerebral ischemia. Mitochondrial COX activity was measured at 550 nm by the reduced cytochrome c method. Data are shown as mean ± SEM (*n* = 6/group). **P < 0.01 *vs.* sham

Compared with the sham group, COX activities were higher in the mitochondria of myocardial tissues of the 6h-ischemia and KDZ groups (both *P* < 0.01). Compared with the 6h-ischemia group, there was no significant change in COX activity in the KDZ group (*P* > 0.05) (Fig. 7).

### KDZ injection enhanced the expressions of COXI and COXIII mRNA in the mitochondria of myocardial tissues after brain ischemia

**Fig. 8:**
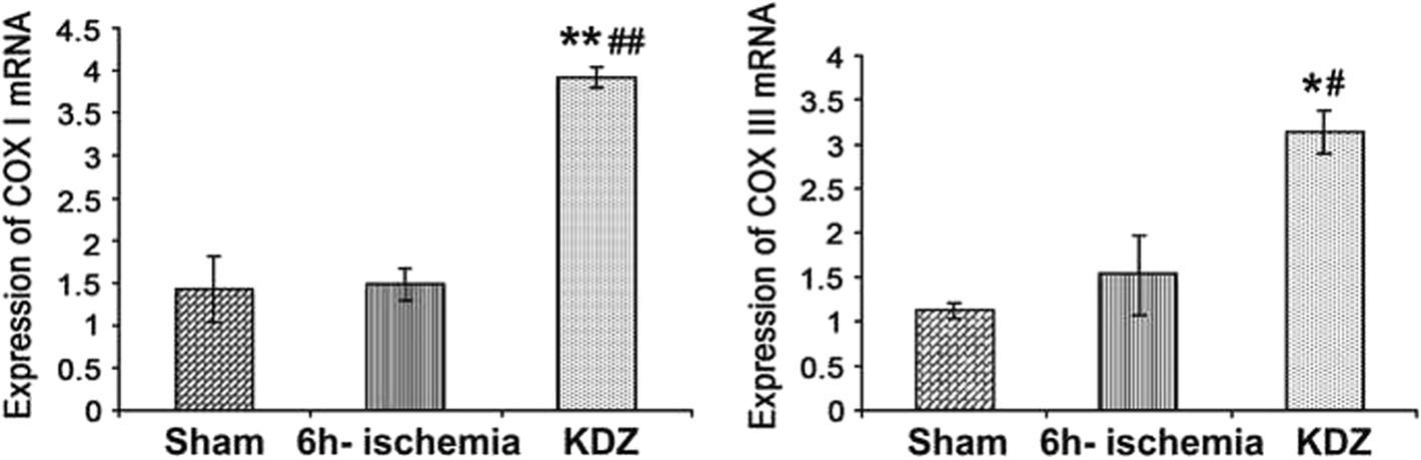
Effects of KDZ injection on the expressions of mitochondrial COX I and COX III mRNA in myocardial tissues of rat models of focal cerebral ischemia. Expressions of mitochondrial COX I and COX III mRNA were determined by RT-PCR. GAPDH was used as control. Data are shown as mean ± SEM (*n* = 6/group). *P < 0.05, **P < 0.01 *vs.* sham; #*P* < 0.05, ##*P* < 0.01 *vs.* 6h-ischemia

RT-PCR results showed that the expressions of COX I and COX III mRNA were not different between the 6h-ischemia and sham groups (both *P* > 0.05). Compared with the 6h-ischemia and sham groups, COX I (both *P* < 0.01) and COX III mRNA (both *P* < 0.05) were significantly higher in the KDZ group (Fig. 8).

## Discussion

Heart failure and ischemic brain stroke are major causes of mortality and disability around the world [25]. Sometimes, the failure of these two organs occurs simultaneously, resulting in a more severe condition [6]. The present study focused on myocardial injury following ischemic stroke using rodent experimental stroke models. Myocardial injury due to brain damage has been previously reported [4, 26]. Although clinical studies have convincingly demonstrated that myocardial injury occurs after cerebral ischemia [4, 26], results from animal studies are still inconsistent, especially at the early stage of acute ischemic stroke [27–29]. The present study strongly suggests that cerebral ischemia could indeed lead to myocardial injury.

Results showed that a 6-h occlusion of the middle cerebral artery of rats resulted in brain functional deficits and edema, with concomitant morphological and biochemical changes in the heart. The myocardial tissue showed histopathological changes that are characteristics of myocardial damage, as supported by higher CK-MB activity and CK and LDH levels in the serum of the rats of the 6h-ischemia group [30, 31]. MF and HF were depressed in ECG recording, but the MF/TF ratio remained unchanged after brain ischemia, suggesting that the balance of the autonomic nervous system was not altered. Increased mitochondrial cytochrome c activity in myocardial tissue might precede the release of cytochrome c into the cytosol and activation of the apoptosis pathways. These findings suggest that acute cerebral ischemia could be a causative event leading to myocardial injury [4, 26].

We further investigated whether KDZ injection is protective against ischemic brain and myocardial injury, and we explored the underlying mechanisms. We found that, in addition to its protection against brain injury, KDZ injection could also alleviate myocardial injury after acute cerebral ischemia. Possibly involved mechanisms include the regulation of oxidative stress and mitochondrial enzyme activity. Measuring by products of lipid peroxidation such as MDA is one of the most widely accepted assays for oxidative stress. In the present study, MDA levels in myocardial tissues were decreased with KDZ injection suggesting alleviated oxidative stress. Meanwhile, KDZ could enhance anti-oxidative defense of myocardial tissues, as evidenced by the increased levels of GSH and SOD activity.

COX is a crucial component of the mitochondrial respiratory chain and is of outmost importance for providing cellular energy [11]. The contribution of COX to ROS production and oxidative stress remains controversial. While increased COX activity has been associated with increased oxidative stress [10, 12], COX inhibition was also found to result in increased susceptibility to oxidative stress and to accelerate mitochondrial apoptosis in response to oxidative stress [13]. KDZ injection was shown to increase COX I and COX III mRNA expression, but the overall mitochondrial COX activity was similar to that of injured animals without KDZ treatment. Whether these changes are relevant to the protective effects of KDZ needs further investigation.

In the present study, to evaluate myocardial function after focal brain ischemia, HRV, which refers to the sinus rhythm within a certain time, was recorded. HRV can be used to quantitatively assess the effects of stroke on the myocardial sympathetic nerve, vagua nerve, and their balance [24]. HRV is a good indicator of the function of the autonomic nervous system of the heart sinus. A reduction in HRV is an independent predictor of cardiac arrhythmias and sudden cardiac death [24, 32]. We showed that MF, HF, and total TF were significantly reduced in the 6h-ischemia group, which could be restored by KDZ injection. The data suggested that the total autonomic activity was significantly impaired by 6-h ischemia and that the function of sympathetic and parasympathetic nerves might be affected after acute cerebral ischemia, which might be associated with secondary heart injury. This finding is in agreement with previous clinical and experimental studies showing reduced MF and HF, and increased MF/HF ratio in patients with cerebral artery occlusion (including insular infarction) [33, 34]. KDZ has the benefits of correcting the myocardial dysfunction after brain ischemia.

Skeletal muscles express 1% of CK-MB and 98% CK-MM, while the cardiac muscle expresses 25–30% CK-MB and 70% CK-MM [35]. Therefore, CK-MB is an appropriate marker of cardiac injury. In the present study, KDZ reversed, in part, the increases in CK-MB, CK, and LDH levels that occurred after acute ischemic stroke. Furthermore, cardiac injury caused by acute ischemic stroke could be attributed, at least in part, to oxidative stress. Similar increases in CK-MB, CK, and LDH levels have been attributed to oxidative stress by a number of studies [30, 31, 36]. A previous study showed that vitamin C (an antioxidant) decreased CK-MB, CK, and LDH levels in mice models of cardiotoxicity, supporting the oxidative stress hypothesis of cardiotoxicity [30].

## Conclusions

The present study suggests that KDZ injection could have a protective effect against cerebral ischemia in rats. It could also alleviate myocardial injury secondary to acute cerebral ischemia in rats. The possible mechanisms involved the regulation of oxidative stress in myocardial tissue after cerebral ischemia in rats. Pharmacological intervention in these aspects may represent a targeted and mechanism-based therapeutic strategy against myocardial injury following cerebral ischemia.

## Abbreviations

ANOVA: Analysis of variance
CK: Creatinine kinase
CK-MB: Creatinine kinase-myocardial band
COX: Cytochrome c oxidase
COX I: Cytochrome c oxidase subunit I
COX III: Cytochrome c oxidase subunit III
ELISA: Enzyme-linked immunosorbent assay
FFT: Fast Fourier transformation
GAPDH: Glyceraldehyde-3-phosphatedehydrogenase
GSH: L-Glutathione
HBFP: Hematoxylin-basic fuchsin picric
HE: Hematoxylin and eosin
HF: High frequency band
HRV: Heart rate variability
KDZ: Kudiezi injection
LDH: Lactate dehydrogenase
LF: Low frequency band
MDA: Malondialdehyde
MF: Medium frequency band
PBS: Phosphate-buffered saline
ROS: Reactive oxygen species
SOD: Superoxide dismutase
TTC: 2, 3, 5-triphenyltetrazo-lium chloride
